# Construction of Shoot Apical Meristem cDNA Yeast Library of *Brassica napus* L. and Screening of Proteins That Interact with the Inflorescence Regulatory Factors BnTFL1s

**DOI:** 10.3390/cimb47010015

**Published:** 2024-12-30

**Authors:** Lingxiong Zan, Haidong Liu, Xutao Zhao, Dezhi Du, Kaixiang Li

**Affiliations:** 1 Academy of Agricultural and Forestry Sciences of Qinghai University, Xining 810016, China; 15003619373@163.com (L.Z.); dahaima@163.com (H.L.); zxtsxbj@163.com (X.Z.); qhurape@126.com (D.D.); 2 Laboratory for Research and Utilization of Qinghai Tibet Plateau Germplasm Resources, Xining 810016, China; 3 Key Laboratory of Spring Rapeseed Genetic Improvement of Qinghai Province, Xining 810016, China; 4 Qinghai Spring Rape Engineering Research Center, Xining 810016, China

**Keywords:** cDNA library, yeast two-hybrid, BnTFL1s, interactive protein, *Brassica napus* L.

## Abstract

The determinate inflorescence trait of *Brassica napus* L. is associated with various desirable agricultural characteristics. *BnTFL1s* (*BnaA10.TFL1* and *BnaC09.TFL1*), which encode the transcription factor *TERMINAL FLOWER 1 (TFL1),* have previously been identified as candidate genes controlling this trait through map-based cloning. However, the mechanism underlying the effects of the BnTFL1 proteins remains unclear. Further, proteins generally interact with each other to fulfill their biological functions. The objective of this study was to construct a cDNA library of the shoot apical meristem (SAM) of *B*. *napus* and screen for proteins that interact with BnTFL1s, to better understand its mechanism of action. The recombination efficiency of the yeast two-hybrid (Y2H) library that we constructed was 100%, with insertion fragment lengths ranging from 750 to 2000 bp and a capacity of approximately 1.44 × 10^7^ CFUs (colony-forming units), sufficient for screening protein interactions. Additionally, the bait vector pGBKT7-BnTFL1s was transformed into yeast cells alongside positive and negative controls, demonstrating no toxicity to the yeast cells and no self-activation. This bait was used to screen the SAM cDNA library of *B. napus*, ultimately identifying two BnTFL1s-interacting proteins: 14-3-3-like protein GF14 omega GRF2. These interactions were verified through one-to-one interaction experiments. This study provides a foundation for further research on the biological functions of the *BnTFL1s* genes and their regulatory role in inflorescence formation in *B. napus*, while providing a reference for studying similar mechanisms in other plants.

## 1. Introduction

Rapeseed (*Brassica napus* L.), also known as canola, is the second largest oilseed crop in the world, after soybean (https://www.ers.usda.gov/topics/crops/soybeans-oil-crops; accessed on 20 August 2024). For rapeseed, efficient mechanized harvesting and high seed yields are highly dependent on its inflorescence architecture. However, natural varieties of *B. napus* exhibit indeterminate inflorescence characteristics, leading to tall plants that are prone to lodging and display inconsistent maturity, making mechanized harvesting challenging and costly. Previous studies have demonstrated that the determinate inflorescence trait of *B. napus* offers several agronomic advantages, including reduced plant height, enhanced lodging resistance, and synchronized maturation, without compromising yields [1,2]. Consequently, investigating the formation of determinate inflorescences in *B. napus* could offer novel approaches for mechanized harvesting and have significant implications for rapeseed breeding.

Previously, our laboratory discovered a determinate inflorescence mutant in the DH line obtained through the microspore culture of (spring *B. napus* × winter *B. napus*). That study found that this trait is controlled by two pairs of independently inherited recessive genes. This led to the identification of the *BnTFL1s* (*BnaA10.TFL1* and *BnaC09.TFL1*), which encode the transcription factor TERMINAL FLOWER 1 (TFL1) and are responsible for the formation of determinate inflorescences in *B. napus* [1,3,4,5]. Moreover, the expression levels of *BnTFL1s* are highest in the shoot apical meristem (SAM) of *B. napus* during the budding stage, and the downregulation of their expression or their deletion will result in determinate inflorescence traits [1,3]. The *TFL1* gene and its homologs have been implicated in inflorescence determination across various plant species [6,7,8]. In rapeseed, many studies have shown that *tfl1* mutants exhibit an earlier flowering time and changes in plant architecture [5,9,10,11]. Despite these findings, the specific protein interactions that regulate TFL1 functions in *B. napus* remain poorly understood.

The mechanism of action of TFL1 is relatively clear in plants such as Arabidopsis and rice. TFL1 is primarily expressed in the central region of the apical meristem. It is a small mobile protein that lacks a DNA-binding domain, and its function is believed to rely on interactions with other transcription factors [12]. In *Arabidopsis*, TFL1 competes with FLOWERING LOCUS T (FT) for binding to the bZIP transcription factor FLOWERING LOCUS D (FD), thereby regulating downstream LEAFY (LFY) and APETALA1 (AP1), which ultimately determines whether the plant flowers [12]. In rice, RCN (TFL1-like protein) competes with Hd3a (FT-like protein) to bind to the GF14c protein, forming a trimer to regulate flowering [7]. Conversely, in cucumber, CsTFL1 does not interact with CsFD or CsGF14. Instead, it interacts with CsNOT2a and binds to CsFD or CsFDP [8]. Consequently, the regulatory pathway of TFL1 in plants is a relatively complex process.

With advancements in functional genomics, yeast two-hybrid (Y2H) and yeast one-hybrid technologies are widely used for detecting protein–protein and protein–DNA interactions in high-throughput proteomic screening [13,14]. High-quality cDNA libraries are essential for analyzing known protein functions and identifying interacting molecules, providing a foundation for constructing protein interaction networks [15]. The library titer, recombination rate, and insert size are indicators of library quality, with a titer threshold of no less than 1.7 × 10^5^ colony-forming units (CFUs) [16,17]. Therefore, constructing a high-quality cDNA library is crucial for studying proteins that interact with target proteins.

To identify proteins related to biological processes, cDNA libraries have been constructed for many plants [17,18], animals [19], and microorganisms [20]. Previous studies have employed forward genetic approaches to identify *BnTFL1s* as candidate genes regulating the inflorescence structure in *B. napus* [1,3,4,5]. To further investigate the role of BnTFL1s in determining the inflorescence architecture, the aim of this study was to construct a high-quality Y2H library from the SAM of *B. napus*. In addition, studies have shown that specific isoforms of 14-3-3 proteins play critical roles in regulating inflorescence development [7,21,22]. During screening, we also found that most candidate interacting proteins were annotated as 14-3-3. Through one-to-one validation, we demonstrated that GRF2 is a key regulatory protein involved in inflorescence formation. This work provides a foundation for further research on the molecular mechanisms of inflorescence development in *B. napus.*

## 2. Materials and Methods

### 2.1. Plant Materials and Growth Conditions

DH Line 2982 of *B. napus* with indeterminate inflorescence, which is the self-pollinated offspring derived from a microspore culture (spring *B. napus* × winter *B. napus)* was used to construct the Y2H library. The conventional varieties cultivated by the Qinghai Academy of Agriculture and Forestry Sciences were used to amplify TFL1 and 14-3-3 from *Brassica rapa L.* and *Brassica oleracea* L. The plants were cultivated in a controlled greenhouse under a 13/11 h light/dark cycle and a temperature regiment of 22/20 °C in Qinghai Province, China (30°55′25″ N, 111°51′24″ E). Young leaves and SAM tissues were collected during the budding stage, immediately flash-frozen in liquid nitrogen, and stored at −80 °C until further analysis.

### 2.2. RNA Extraction and cDNA Library Construction

Total RNA was extracted from the collected plant tissues using the TRIzol Reagent kit (Invitrogen, USA), following the manufacturer’s instructions. mRNA isolation and purification were performed using the Oligotex mRNA Midi Kit (Invitrogen, USA). RNA quality was assessed by spectrophotometry to confirm its integrity and suitability for subsequent cDNA synthesis. The CloneMiner II cDNA library construction kit (Invitrogen, Carlsbad, CA, USA) was used to synthesize the first strand of cDNA, followed by the synthesis of double-stranded cDNA, which was then ligated using the attB1 recombinant adapter. This cDNA was then fractionated and integrated into the pDONR222 vector via a BP reaction using the BP Clonase ^®^ II (Thermo Fisher Scientific, Waltham, MA, USA) enzyme mix, and subsequently transformed into *Escherichia coli* DH10B cells. The primary library of bacterial liquid was produced by shaking the transformation medium.

### 2.3. Library Capacity and Insert Length Identification

To assess the quality of the bacterial library, a 100-fold dilution of a 10 μL aliquot of the bacterial solution was spread on an LB plate containing kanamycin. Library capacity was calculated based on the CFU formula, as follows: library titer (CFU/mL) = number of clones on the plate/volume of bacterial solution coated on the plate (50 μL) × dilution factor (100) × 10^3^ μL; the total library capacity was derived from the titer multiplied by the total volume of the bacterial solution. Twenty-four clones were randomly selected and identified via polymerase chain reaction (PCR) analysis, and the recombination rate and the lengths of the insert fragments were determined by visualizing PCR products via agarose gel electrophoresis.

### 2.4. Yeast Secondary Library Construction

The primary library that was verified as qualified was shaken overnight in broth culture medium, and plasmids were extracted using a reagent kit. The plasmid solution was diluted to 300 ng/µL and used for LR recombination with 1 μL of the pGADT7-DEST vector. The resulting recombinant product was transformed into *E. coli* DH10B cells to generate a secondary bacterial library. The identification of the secondary library capacity, recombination rate, and insertion fragment length were carried out using the same method as that for the primary library.

### 2.5. Construction of theY2H Bait Vector pGBKT7-BnTFL1s

The bait vector pGBKT7 was linearized using EcoR I and BamH I, and *BnTFL1s* genes were amplified using the following primers: BnTFL1s-F (5′-CCATGGAGGCCAGTGAATTCATGGAGAATATGGGAACTAGAGTGA-3′) and BnTFL1s-R (5′-ATCTGCAGCTCGAGCTCGATGGATCCTTAACGTCTGCGAGATGCGG-3′). PCR amplification was performed using Phusion polymerase under standard conditions (98 °C for 1 min, followed by 35 cycles of 98 °C for 10 s, 58 °C for 30 s, and 72 °C for 1 min, with a final extension at 72 °C for 10 min). The PCR product was recombined with the linearized pGBKT7 vector using Vazyme’s ClonExpress II One-Step Cloning Kit. The recombinant pGBKT7-BnTFL1s plasmid was transformed into *E. coli* DH5α cells via the heat shock method. Positive clones were identified using the universal primers T7 and 3BD of the pGBKT7 vector, and the plasmid was extracted after sequence verification. The pGBKT7-BnTFL1s vector was thereby constructed.

### 2.6. Detection of Bait Vector Toxicity and Self-Activation

The pGBKT7-BnTFL1s recombinant plasmid was co-transformed with the pGADT7 prey vector into Y2H Gold yeast cells using the polyethylene glycol/lithium acetate method (LiAc). The interactions between pGBKT7-53 and pGADT7-T and between pGBKT7-Lam and pGADT7-T were used as the positive and negative control, respectively. The transformation product was resuspended and diluted in a 0.9% NaCl solution, and 80 μL of the bacterial solution was spread onto SD/-Trp/-leu/X-α-gal (DDO/X) and SD/-Trp/-leu/-His/-Ade/X-α-gal/AbA (QDO/X/A) plates and incubated at 30 °C for 3–5 days. The colony size and color were observed to determine whether the bait vector exhibited toxicity or self-activation.

### 2.7. Screening for BnTFL1s-Interacting Proteins

The bait vector pGBKT7-BnTFL1s (5 μL) and the Secondary Library Plasmid of *B. napus* SAM (10 μg) were co-transformed into Y2H Gold yeast cells using the LiAc transformation method. The conversion solution was suspended in 0.9% NaCl solution to a total volume of 6 mL, and all cells were coated on DDO/X plates for preliminary screening. Each plate was coated with 80 μL, approximately 80 pieces; then, they were cultivated at a constant temperature of 30 °C for 3−5 days and grown with monoclonal antibodies to a size of 1–2 mm. The blue clones that formed on the DDO/X plates during this preliminary screen were transferred to QDO/X/A plates for secondary, high-stringency screening. These plates were also incubated at 30 °C for 3–5 days. Finally, clones that could grow normally and appeared blue were selected for a comparison with positive controls for PCR detection and sequencing.

### 2.8. Identification and Validation of Candidate Interacting Proteins

A single colony was inoculated into SD/-Trp/-Leu medium and cultured with shaking at 30 °C for 48 h. Yeast plasmid extraction was then performed using the yeast plasmid extraction kit. A 5 μL sample of the yeast plasmid was transformed into DH5α competent cells via the heat shock method. After transformation, the cells were spread onto LB plates containing ampicillin and incubated upside down at 37 °C for approximately 16 h. A single colony was selected from the plate, inoculated into LB liquid medium with ampicillin, and cultured overnight at 37 °C with shaking at 260 rpm. Following this, plasmid extraction was performed. Genes encoding the correctly interacting proteins were sequenced by Shanghai Sangon Biotechnology, and the sequences that were successfully sequenced without frameshift mutations were subjected to a BLAST search to obtain the complete open reading frame sequence of the candidate interacting protein using the NCBI database. Simultaneously, the function of each candidate protein was predicted, with a focus on selecting proteins related to inflorescence development for one-to-one validation. Both the prey and bait plasmids pGBKT7-BnTFL1s were co-transformed into the Y2H Gold yeast cells using the LiAc transformation method, and the transformed cells were plated onto DDO/X and QDO/X/A plates to observe the growth status of the colonies, to verify the screening results.

## 3. Results

### 3.1. Total RNA Extraction, mRNA Purification, and Double-Stranded cDNA Synthesis

**Figure 1:**
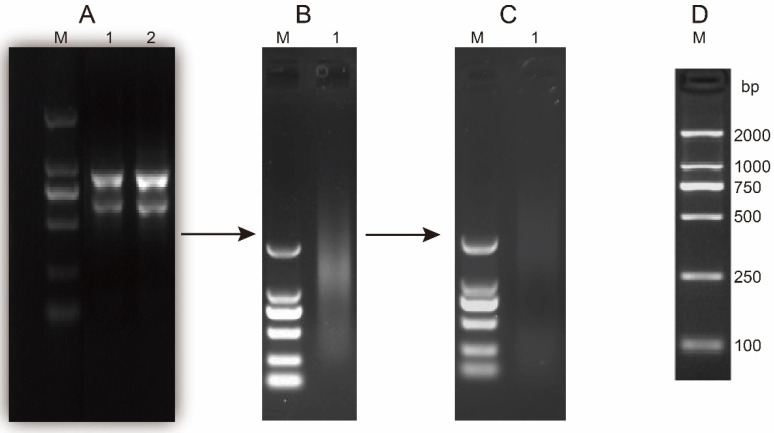
Process of total RNA extraction, mRNA purification, and double-stranded-complementary DNA (ds-cDNA) synthesis. (**A**) Total RNA was extracted from the shoot apical meristem (SAM) of *B. napus*. (**B**) mRNA was isolated from total RNA. (**C**) ds-cDNA was synthesized. (**D**) DL 2000 DNA Marker, 1 and 2: RNA, mRNA or cDNA from *B. napus* SAM.

Total RNA was extracted from the SAM of the *B. napus* indeterminate inflorescence 2982 line to construct a Y2H library. The OD260/OD280 ratio of total RNA was 2.12, and the OD260/OD230 ratio was 1.80, indicating satisfactory RNA purity. Ribosomal 28S and 18S bands were visible on a 1% agarose gel, with the 28S band brighter than the 18S band (Figure 1A), confirming the integrity of the RNA sample. The mRNA electrophoresis (Figure 1B) showed a diffuse band, with the brightest section representing a higher molecular weight range, signifying successful mRNA isolation and purification. Subsequent reverse transcription generated the first and second strands of cDNA (Figure 1C).

### 3.2. Construction and Identification of the B. napus SAM Y2H Library

**Figure 2:**
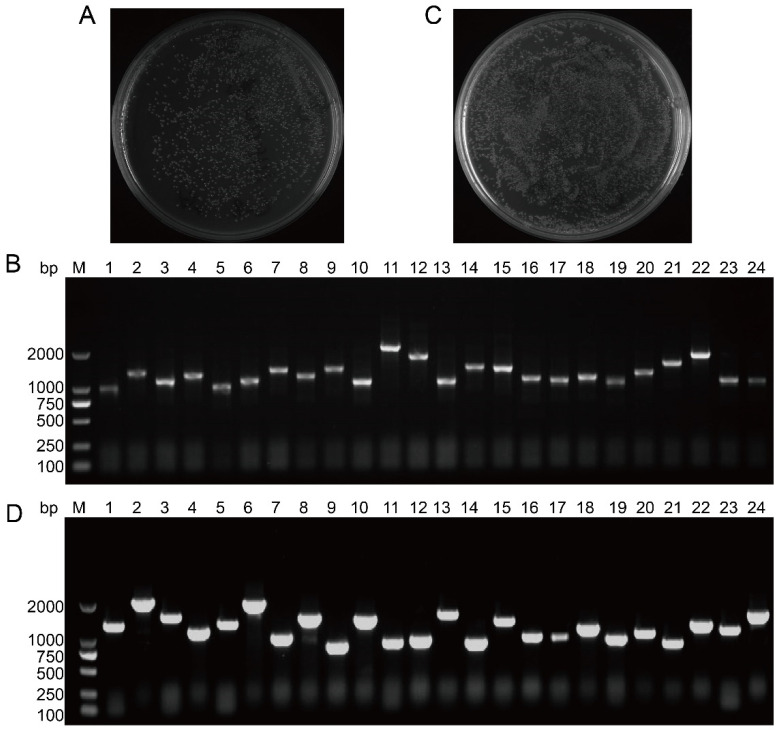
Identification of library capacity and insert length for yeast two-hybrid (Y2H) libraries. (**A**) Identification of primary library capacity. (**B**) Identification of insert length and recombination rate for primary library. M: DL 2000 DNA Marker; 1–24: PCR products of 24 colonies. (**C**) Identification of secondary library capacity. (**D**) Identification of insert length and recombination rate for secondary library. M: DL 2000 DNA Marker; 1–24: PCR products of 24 colonies.

cDNA was ligated into the pDONR222 vector via homologous recombination, resulting in a three-frame cDNA library with a bacterial population of 2.6 × 10^6^ CFU/mL and a total of approximately 1300 clones. The library capacity was calculated as approximately 1.04 × 10^7^ CFU (Figure 2A). Colony PCR analysis of 24 random clones revealed a single band between 1000 and 2000 bp, with a recombination rate of 100%, meeting the standards of the primary library (Figure 2B). After plasmid extraction, the secondary library was constructed with a bacterial count of 3.6 × 10^6^ CFU/mL and a capacity of 1.44 × 10^7^ CFU(Figure 2C). All 24 clones from the secondary library exhibited a 100% recombination rate, with insertion sizes ranging from 750 to 2000 bp and demonstrating abundant polymorphism (Figure 2D). These results suggest that the Y2H library was of high quality and was suitable for further screening.

### 3.3. Construction of Bait Vector pGBKT7-BnTFL1s

**Figure 3:**
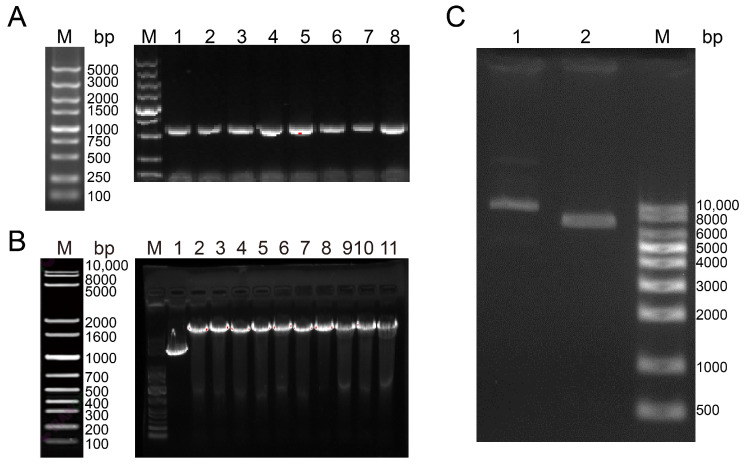
Construction of the bait vector pGBKT7-BnTFL1s. (**A**) Amplification of *BnTFL1s* genes from the cDNA of the *B. napus* shoot apical meristem (SAM); lanes 1–8: PCR products from eight individual colonies. (**B**) Results of double enzyme digestion of the pGBKT7 vector; lane 1: circular pGBKT7 vector; lane 2–11: pGBKT7 vector digested with EcoRI and BamHI. (**C**) Double digestion results of the bait vector pGBKT7-BnTFL1s; lane 1: bait vector pGBKT7-BnTFL1s; lane 2: bait vector pGBKT7-BnTFL1s digested with EcoRI; and BamHI.

The full-length *BnTFL1s* genes (537 bp) were amplified successfully using 2982 cDNA, as confirmed via gel electrophoresis (Figure 3A). The pGBKT7 plasmid was linearized through EcoRI and BamHI digestion (Figure 3B), and the genes were ligated into the vector via homologous recombination. After transformation into *E. coli* DH5α, successful construction of the pGBKT7-BnTFL1s bait vector was confirmed by performing restriction digestion (Figure 3C) and sequencing.

### 3.4. Toxicity and Self-Activation Activity of pGBKT7-BnTFL1s

**Figure 4:**
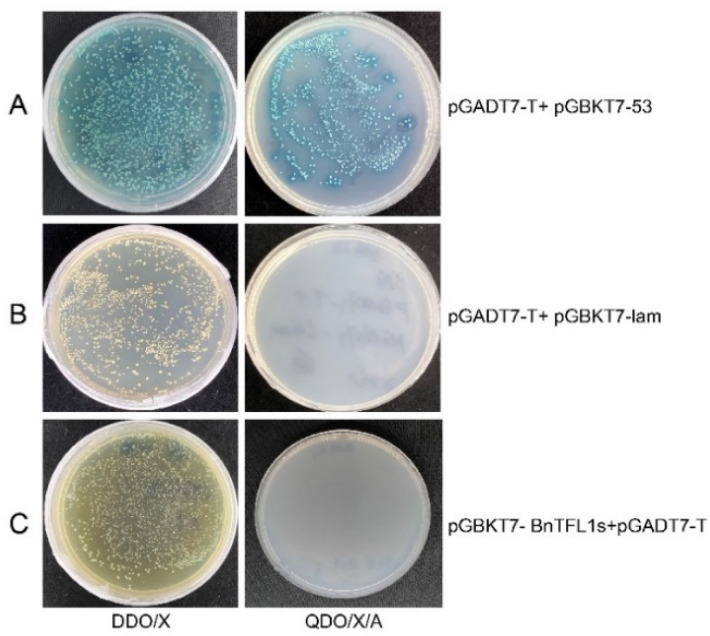
The bait plasmid pGBKT7-BnTFL1s does not exhibit self-activation. (**A**) Co-transformed pGBKT7–53 and pGADT7-T as a positive control. (**B**) Co-transformed pGBKT7-Lam and pGADT7-T as a negative control. (**C**) Co-transformed pGBKT7-BnTFL1s and pGADT7-T for self-activation verification. DDO/X: SD/-Trp/-leu/X-α-gal culture media; QDO/X/A: SD/-Trp/-leu/-His/-Ade/X-α-gal/AbA culture media.

The bait plasmid pGBKT7-BnTFL1s and empty pGBKT7 vector were transfected into Y2H Gold yeast cells and plated on SD/-Trp medium. Following a 5-day incubation at 30 °C, the growth and size of the yeast colonies were evaluated. The number and size of the colonies from both strains were comparable, indicating that the pGBKT7-BnTFL1s bait vector is not toxic to yeast cells. Meanwhile, the positive control, negative control, and self-activation detection constructs were each co-transformed into Y2H yeast competent cells. The transformed yeast cells were then spread on DDO/X and QDO/X/A plates. The combination of pGBKT7-53 and pGADT7-T resulted in blue clone growth in both media (Figure 4A). In contrast, the combination of pGBKT7-Lam and pGADT7-T demonstrated clonal growth in the DDO/X medium; however, no blue color was produced, and no clones grew on the QDO/X/A medium, confirming the success of the negative control experiment (Figure 4B). In addition, when the experimental groups pGBKT7-BnTFL1s and pGADT7 were transformed together with DDO/X, no blue clones were generated, and there was no growth on QDO/X/A, which is consistent with the results of the negative control. This also proves that the bait vector pGBKT7-BnTFL1s does not self-activate in yeast (Figure 4C) and could be directly used for subsequent screening work.

### 3.5. Screening of BnTFL1s-Interacting Proteins

**Figure 5:**
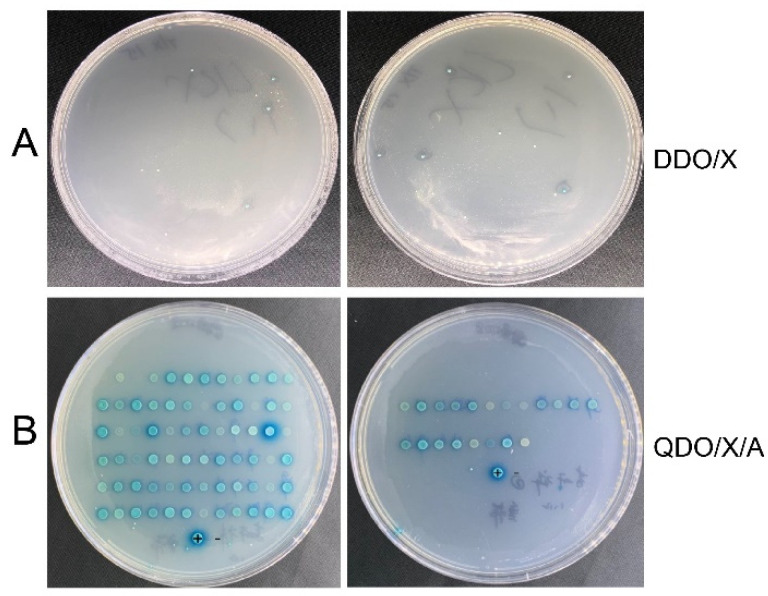
A total of 62 clones can grow normally and produce blue color on QDO/X/A plates. (**A**) Result of initial screening of positive clones on DDO/X plates. (**B**) Growth of 92 blue clones on QDO/X/A plates. The symbols ‘+’ and ‘−’ indicate the positive and negative controls, respectively.

The pGBKT7-BnTFL1s bait plasmid and *B. napus* SAM secondary library of plasmids were co-transformed into competent Y2H Gold yeast cells. Following transformation, the cells were resuspended in a 0.9% NaCl solution and spread onto DDO/X plates for preliminary screening. Here, 92 blue colonies were identified, with representative images shown in Figure 5A. Based on a subsequent calculation, the yeast conversion efficiency exceeded 2 × 10^5^ CFU/μg, producing 2 × 10^6^ CFU of transformants. The 92 blue clones from the DDO/X plates were then transferred to QDO/X/A plates for more rigorous screening, where 62 blue clones were similar to the positive clones, whereas the other 30 did not grow normally or produce a blue color, indicating that these clones did not harbor candidate proteins for interactions with BnTFL1s (Figure 5B).

From these 62 blue colonies, 20 individual colonies that were successfully sequenced without frameshift mutations were selected through PCR sequencing (Table 1). After BLAST analysis and functional annotation, several proteins unrelated to inflorescence development (AD-9, 13, 19, 26, 31, 33, and 37) were excluded, and half of the clones with the same annotation were selected for analysis. Eight clones were ultimately selected and inoculated into liquid SD/-Trp/-Leu medium for expansion and yeast plasmid extraction. The plasmids were then transformed into DH5α competent cells, followed by extraction and sequencing (Table 2). Clones 29, 35, and 36 showed inconsistencies with the initial PCR sequencing results. Ultimately, five correctly sequenced candidate interacting proteins were identified.

### 3.6. One-to-One Verification of Candidate Proteins Interacting with pGBKT7-BnTFL1s

**Figure 6:**
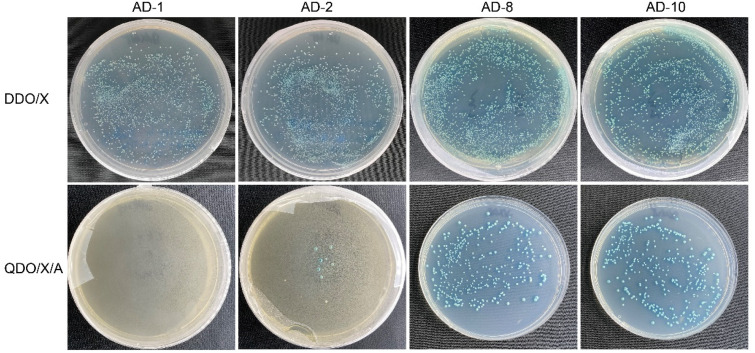
Results of partial one-to-one interaction verification of candidate proteins interacting with pGBKT7-BnTFL1s.

**Figure 7:**
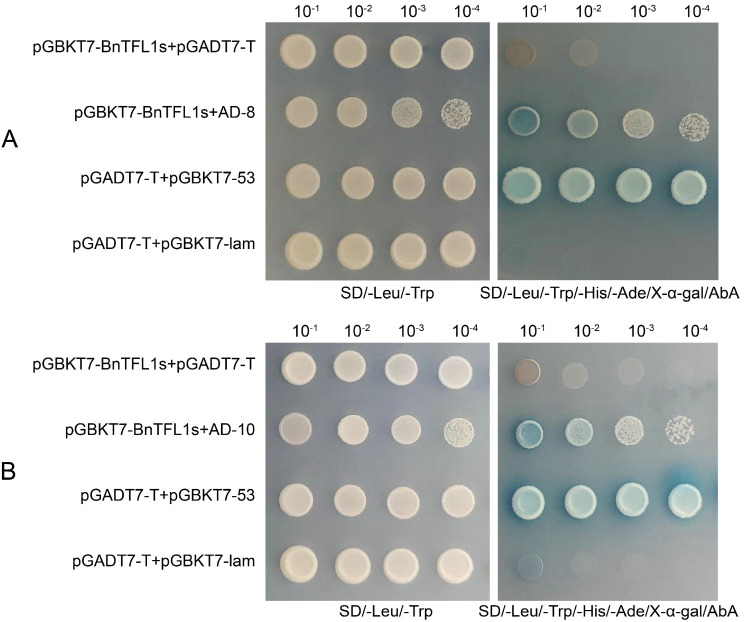
One-to-one interaction validation. (**A**) AD-8 interacted with pGBKT7-BnTFL1s in one-to-one interaction validation. (**B**) AD-10 interacted with pGBKT7-BnTFL1s in one-to-one interaction validation.

Five candidate interacting proteins were validated through one-to-one interaction testing as performed with pGBKT7-BnTFL1s. The results demonstrated that three positive clones, AD-2, AD-8, and AD-10, interacted with the BnTFL1s. Notably, the interaction between AD-2 and BnTFL1s was weak, whereas the interactions involving AD-8 and AD-10 with BnTFL1s were stronger (Figure 6). The results of these interactions are summarized in Table 2. Because studies showed an interaction between TFL1 and 14-3-3 proteins in rice and *Arabidopsis* [7,23], AD-8 and AD-10 were annotated as *Arabidopsis* 14-3-3-like protein GF14 omega GRF2, related to the regulation of flowering and inflorescence development. The results of the plate validation based on AD-8 (Figure 7A) and AD-10 (Figure 7B) were consistent with the results of the one-to-one interaction analysis. Therefore, we propose that this protein might functionally interact with BnTFL1s, and we intend to focus on studying it in future research.

### 3.7. Analysis of TFL1 and 14-3-3 Interaction in B. rapa L. and B. oleracea L.

**Figure 8:**
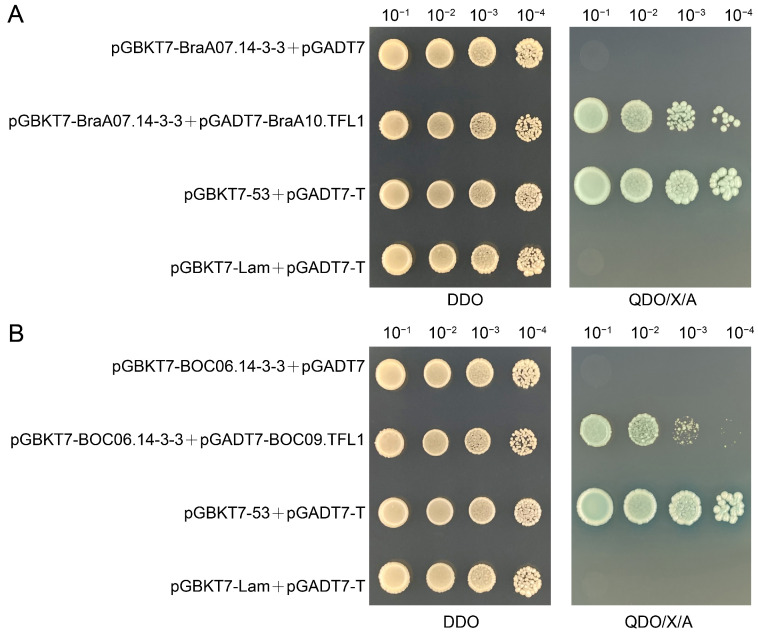
TFL1 interacts with 14-3-3 in both *Brassica rapa* L. and *Brassica oleracea* L. (**A**) BraA10.TFL1 interacted with BraA07.14-3-3 in *B. rapa.* (**B**) BOC06.14-3-3 interacted with BOC09.TFL1 in *B. oleracea.*

To investigate the interaction between TFL1 and 14-3-3 in the two basic varieties of *B. napus*, specific primers were designed to amplify the relevant genes in *B. rapa* and *B. oleracea* and used to construct bait or prey vectors. We followed the one-to-one validation method described in this study and conducted Y2H experiments. The results showed that BraA10.TFL1 interacted with BraA07.14-3-3 in *B. rapa* (Figure 8A) and that BOC06.14-3-3 interacted with BOC09.TFL1 in *B. oleracea* (Figure 8B). These experiments confirmed that these two proteins also interact in diploid basal species before the formation of tetraploid *B. napus.*

## 4. Discussion

The Y2H system offers distinct advantages for studying protein–protein interactions. First, it is applicable across various tissues and organs, making it suitable for large-scale screening and library construction to identify interacting proteins associated with known proteins from diverse species. Second, during the library screening process, the gene sequence encoding the interacting protein can be obtained, thereby eliminating the cumbersome steps of protein extraction and purification that are required in other in vitro detection methods. Additionally, the fusion protein interacts within yeast cells, allowing the protein to maintain a degree of natural folding, which closely approximates physiological conditions. Consequently, cDNA libraries have been established for numerous species to facilitate the screening of proteins that interact with known proteins [17,18,19,20]. In this study, we constructed a high-quality *B. napus* SAM cDNA library and screened for proteins that interact with BnTFL1s.

TFL1 and its related proteins interactions play a crucial role in regulating the plant flowering time and inflorescence architecture, with its mechanism of action extensively studied across various plant species (Table S1) [7,8,12,23,24,25,26,27,28,29,30]. In *Arabidopsis* and potato, TFL1 and FT compete for binding to the FD protein to regulate downstream floral meristem genes, performing opposing functions [12,31]. In rice, RCN and Hd3a competitively bind to the 14-3-3 protein, forming a trimer that either inhibits or activates the transcriptional regulation of OsMADS15, a homolog of AP1 in rice, affecting the flowering time [7,21]. In apple, both MdTFL1 and MdFT interact with four Md14-3-3 proteins in the cytoplasm, subsequently translocating to the nucleus to associate with FD proteins, thereby jointly regulating the expression of downstream flowering genes [22]. Conversely, in cucumber, CsTFL1 inhibits the expression of CsLEY and CsAP1 by forming a complex with CsNOT2a and CsFDP, which maintains the indeterminate growth of cucumber [8]. This variation in the mechanism of action of TFL1 across different plants highlights the necessity of studying its interaction mode in *B. napus*. This study demonstrates that BnTFL1s interact with 14-3-3ω GRF2 in *B. napus*. However, in our study, no interaction between the BnTFL1s and BnFD was identified, which could suggest that in *B. napus*, this might require the involvement of other intermediate proteins or specific experimental conditions.

Our research group was the first to report that BnTFL1s is a key factor in regulating the determinate inflorescence of *B. napus*, as demonstrated through experiments including map-based cloning, expression pattern analysis, and genetic transformation [1,3,4,5]. However, since TFL1 requires interactions with other proteins to function, proteins that interact with BnTFL1s were screened in this study to facilitate further research. Wang et al. selected and confirmed the interaction between BnaA05.GF14nu and BnaA10.TFL1 through a homology comparison with the *Arabidopsis thaliana* 14-3-3 protein [32]. In contrast, in this study, the interaction between GRF2 and BnTFL1s was screened by establishing a Y2H library using our own materials, which may yield more reliable and convincing results. This finding provides a foundation for our subsequent investigation into the molecular mechanisms through which BnTFL1s regulate indeterminate inflorescence and is of great significance for studies on inflorescence architecture regulation in *B. napus*.

Additionally, we demonstrated that the BnTFL1s interact specifically with the 14-3-3 omega GRF2 isoform but not with the kappa GRF8 or epsilon GRF10 isoforms (Table 2). This indicates that isoform-specific 14-3-3 proteins specifically bind to distinct partners and participate in various biological processes. Similar reports based on many plants can prove this viewpoint. The *Arabidopsis* 14-3-3λ/κ protein interacts with phosphorylated photosensitizer interacting factor 3 (PIF3) to promote PIF3 degradation, thereby positively regulating plant photomorphogenesis [33]. The interaction between rice OsGF14c and BZR1 reduces the accumulation of BZR1, inhibits the expression of downstream regulatory genes, and thus affects the BR signaling pathway [34]. Further, the phosphorylation of rice OsGF14e by the calcium-dependent protein kinase OsCPK21 enhances the response of the plant to ABA [35]. Rice OsGF14h inhibits the ABA signaling pathway through interactions with the transcription factors HOX3 and VP1 [36]. In addition, rice OsGF14b interacts with OsPLC1 and promotes its activity and stability, thereby improving the salt tolerance of rice [37]. The Gf14f and Gf14g proteins in rice can interact with the *Xanthomonas oryzae* protein (Xop) and participate in regulating signal transduction related to plant innate immunity [38]. Finally, wheat TaGRF6-A actively regulates salt tolerance through an interaction with the MYB transcription factor TaMYB64 [39].

Meanwhile, we also cloned TFL1 and 14-3-3 from two basic varieties of *B. napus*, *B. rapa*, and *B. oleracea*, and conducted Y2H experiments. These experiments confirmed that both genes interact in the diploid species. This led us to speculate that gene interactions prior to the formation of *B. napus* are genome-specific (A-to-A and C-to-C). During the natural hybridization and chromosomal duplication processes that resulted in tetraploid *B. napus*, not only did the number of genes increase, but gene interactions also became more complex. This likely led to the emergence of cross-genome interactions between the A and C genomes.

## 5. Conclusions

In this study, we aimed to identify factors that interact with the inflorescence regulatory factor BnTFL1s by constructing a high-quality Y2H library of *B. napus*. An analysis of the quality of the library revealed a recombination efficiency of 100%, an insert length ranging from 750 to 2000 bp, and a library capacity of approximately 1.44 × 10^7^ CFU. Additionally, the bait pGBKT7-BnTFL1s was used to screen the SAM cDNA library of *B. napus*, ultimately identifying two BnTFL1s-interacting proteins, the 14-3-3-like protein GF14 omega GRF2. These results were further validated through one-to-one interaction experiments. These findings suggest that a 14-3-3 protein, particularly the 14-3-3 omega GRF2 isoform, may play a role in the BnTFL1s-mediated regulation of *B. napus* inflorescence trait. In summary, this study establishes a foundation to further explore the molecular mechanisms underlying the inflorescence regulatory factors BnTFL1s in *B. napus* and provides insights into the molecular mechanisms underlying the functions of TFL1 in other plants.

## Figures and Tables

**Table 1:** Analysis of sequencing results of 20 candidate positive clones.

AD Number	Putative Function	Gene Name
1, 3, 5, 29, 36, 38, 39	14-3-3-like protein GF14 lambda [*B. rapa*]	XP_009125300.1
2	mitochondrial outer membrane protein porin 3 [*B. napus*]	XP_013662361.1
6	14-3-3-like protein GF14 psi [*B. napus*]	XP_022552879.1
35		
8	unnamed protein product, partial [*B. oleracea*]	VDD64795.1
9	protein phosphatase 2C 70 isoform X1 [*B. rapa*]	XP_009120903.1
10	unnamed protein product, partial [*B. oleracea*]	VDD62767.1
13	mitochondrial outer membrane protein porin 3-like [*B. oleracea*]	XP_013606965.1
19	pentatricopeptide repeat-containing protein At2g17670 [*B. napus*]	XP_013693355.1
26	mitochondrial outer membrane protein porin 1-like [*B. oleracea*]	XP_013637592.1
28	hypothetical protein F2Q69_00054638, partial [*B. cretica*]	KAF3485538.1
31	protein LHCP TRANSLOCATION DEFECT-like [*B. oleracea*]	XP_013638801.1
33	heat stress transcription factor A-4a [*B. rapa*]	XP_009136968.1
37	glyceradehyde-3-phosphate dehydrogenase, partial [*B. rapa*]	BAJ10476.1

**Table 2:** Analysis of one-to-one interaction results of five candidate positive clones.

AD	Gene Bank	(TDO/X)	(QDO/X/A)	Arabidopsis Homologous
1	BnaC09G0088500ZS/BnaA09G0091600ZS	growth	no growth	14-3-3-like protein GF14 kappa GRF8
8	BnaC06G0440000ZS/BnaA07G0374100ZS	growth	blue	14-3-3-like protein GF14 omega GRF2
10	BnaA07G0235600ZS/BnaC06G0256100ZS	growth	blue	14-3-3-like protein GF14 omega GRF2
28	BnaC07G0182400ZS/BnaA07G0123900ZS	growth	no growth	14-3-3-like protein GF14 epsilon GRF10
2	XP_013662361.1	growth	blue	

## Data Availability

The original contributions presented in this study are included in the article/Supplementary Material. Further inquiries can be directed to the corresponding author(s).

## Supplementary Materials

The following supporting information can be downloaded at: https://www.mdpi.com/article/10.3390/cimb47010015/s1, Table S1: Identified interactions in plants and their potential roles in inflorescence development.
