# Supplementary material for: Construction of Shoot Apical Meristem cDNA Yeast Library of Brassica napus L. and Screening of Proteins That Interact with the Inflorescence Regulatory Factors BnTFL1s
Source: Curr Issues Mol Biol. 2024 Dec 30;47(1):15. doi: 10.3390/cimb47010015 (PMC11764432; doi:10.3390/cimb47010015)
Supplement: Supplementary file 1 [file cimb-47-00015-s001.zip › cimb-3352460-supplementary.pdf]

Table S1 Identified interactions in plants and their potential roles in inflorescence development

Species	Target gene	Interacting protein	Potential roles in inflorescence development	Reference
Arabidopsis	TFL1/FT	FD	TFL1 competes with FT for binding to FD and ultimately delays or accelerates flowering	12
Rice	RCN/Hd3a	14-3-3	Hd3a forms a ternary "flowering activation complex" (FAC) with the 14-3-3 protein and OsFD1, which can induce the transcription of OsMADS15 and induce flowering	7
Medicago truncatula	MtFDa	MtFTa1	MtFDa physically interacts with MtFTa1 to promote MtSOC1a expression and regulate the flowering time and inflorescence development	24
Arabidopsis	RPT2a	MET1	Their interaction can facilitate the degradation of MET1, thereby regulating the DNA methylation level of *TFL1* promoter and affecting *TFL1* expression, ultimately regulating inflorescence meristem activity and the inflorescence configuration	25
Potato	StAST1	StSP6A	StSP6A interacts with StAST1 causing transcriptional reprogramming at stolon subapical apices	26
Arabidopsis	BRC1	TIE1	BRC1 interacts with TIE1 regulating stem and branch branching	27
Cucumber.	CsTFL1/ CsFT	CsNOT2a	CsTFL1 competes with CsFT for interactions with CsNOT2a–CsFDP, thereby inhibiting deterministic growth and apical flower formation in cucumber.	8
Apple	MdGF14s	MdTFL1 and MdFT	Four subtypes of GF14 (MdGF14a, MdGF14d, MdGF14i, and MdGF14j) interact with MdTFL1 and MdFT to participate in flowering regulation	23
Cotton	GhMFT1and GhMFT2	GhFD	GhMFT1 and GhMFT2 interact with GhFD to regulate cotton seed germination	28
Pea	VEG2	FT	VEG2 functions synergistically with multiple FT proteins to regulate the expression of downstream target genes, promotes cross-regulation within the FT gene family, and plays a role in the development of compound inflorescences in peas	29
Maize	BIF2	BA1	BIF2 promotes maize axillary meristem initiation through an interaction with BA1	30
